# Atmospheric Room Temperature Plasma Pretreatment Enhances Pulsed Electric Field Extraction of Phenolic Compounds from Hemp Leaves: Response Surface Modeling and Stability Assessment in a Model Vinegar System

**DOI:** 10.3390/molecules31173127

**Published:** 2026-09-07

**Authors:** Eleni Bozinou, Vassilis Athanasiadis, Stavros I. Lalas, Arhontoula Chatzilazarou

**Affiliations:** 1 Department of Wine, Vine & Beverage Sciences, University of West Attica, Agiou Spyridonos Street, 12243 Athens, Greece; ebozinou@uniwa.gr; 2 Department of Food Science and Nutrition, University of Thessaly, Terma Nikou Temponera Street, 43100 Karditsa, Greece; vaathanasiadis@uth.gr

**Keywords:** *Cannabis sativa* leaves, non-thermal processing, electroporation-assisted extraction, plasma-activated materials, phenolic profile, antioxidant capacity, HPLC–DAD analysis, acidic food matrices, polyphenol stability, acetic acid systems

## Abstract

The integration of emerging non-thermal technologies for the valorization of plant bioactive compounds is gaining increasing attention in food and natural product research. In this study, Atmospheric Room Temperature Plasma (ARTP) pretreatment combined with pulsed electric field (PEF) extraction was investigated as an innovative strategy to enhance the recovery of bioactive compounds from *Cannabis sativa* L. (cv. Futura 75) leaves. Response Surface Methodology (RSM) was employed to identify the best conditions within the investigated range for the ARTP process based on total phenolic content (TPC), DPPH^•^ radical scavenging activity, and ferric reducing antioxidant power (FRAP). Under the best conditions within the investigated range (16.6 mm electrode–sample distance, 1.6 mm sample thickness, 70% plasma intensity, 10 L min^−1^ nitrogen flow, and 1 min treatment), ARTP pretreatment increased TPC, FRAP, and DPPH values by 23.0%, 68.6%, and 46.1%, respectively, compared with PEF extraction alone. HPLC–DAD analysis confirmed that ARTP pretreatment selectively enhanced the recovery of the identified phenolic compounds, particularly protocatechuic acid and luteolin-7-*O*-glucoside, supporting the observed increases in antioxidant capacity. The extract obtained under the best conditions within the investigated range was incorporated into a model vinegar system (6% *v*/*v* acetic acid), and its stability was evaluated over 28 days by investigating the effects of extract concentration, storage temperature, and light exposure on TPC and antioxidant capacity. Elevated temperature (40 °C) and light exposure accelerated antioxidant degradation, whereas the highest extract concentration (1.2 g L^−1^), storage at ambient temperature (20 °C), and absence of light resulted in improved stability. These findings demonstrate that the combined ARTP–PEF approach enhances phenolic extraction from *C. sativa* leaves and yields extracts with improved stability, highlighting its potential for developing functional ingredients for acidified food systems.

## 1. Introduction

*Cannabis sativa* L., widely recognized as Indian hemp, is an annual herbaceous species that has been known since antiquity, particularly in Central Asia, including India and China [1]. Throughout history, its cultivation and use have varied across different civilizations and historical periods. The plant has served as a valuable source of fiber, food, edible oil, and medicinal preparations [2], while it has also been associated with religious ceremonies and recreational use [1,3]. Its biological and therapeutic properties are mainly attributed to the presence of numerous bioactive constituents, including cannabinoids, terpenoids, flavonoids, and alkaloids [4]. Among these, cannabinoids represent the most characteristic group, comprising more than one hundred structurally related terpenophenolic compounds that exhibit diverse biological activities [5] and are predominantly accumulated within the glandular trichomes of female flowers [2]. The principal cannabinoids include Δ9-tetrahydrocannabinol (Δ9-THC), responsible for the plant’s psychoactive effects [6], and cannabidiol (CBD), a non-psychoactive cannabinoid that is particularly abundant in industrial hemp varieties [7]. Besides cannabinoids, hemp is also rich in polyphenolic compounds such as gallic acid, catechin [8], caffeic acid, p-coumaric acid, ferulic acid, luteolin-7-*O*-glucoside, apigenin, kaempferol-3-*O*-glucoside, apigenin-7-glucoside, myricitrin, rutin, chlorogenic acid, and quercetin-3-glucoside [9]. These phenolic compounds contribute substantially to the antioxidant properties of hemp [10], which have been extensively evaluated using assays such as DPPH and FRAP [11,12].

Considerable research has recently focused on developing innovative extraction technologies capable of overcoming the limitations associated with conventional methods for recovering plant-derived bioactive compounds. Among these emerging approaches, pulsed electric field (PEF) technology has attracted increasing attention as an effective extraction tool [13]. PEF is a green, non-thermal processing technique originally developed for food preservation, where short electrical pulses are applied to inactivate microorganisms while minimizing quality deterioration [14]. Typically, the process employs pulses of very short duration, ranging between 100 ns and 1 ms, with voltages from 1 to 3 kV [15]. Beyond microbial control, PEF has been increasingly used to improve extraction efficiency [13], as well as to facilitate diffusion, osmotic dehydration, pressing, and drying operations involving food materials and agro-industrial by-products [16]. An additional advantage of this technology is its ability to reduce the detrimental effects associated with conventional thermal treatments [14,17]. By inducing electroporation of cell membranes, PEF enhances the release of intracellular compounds and is therefore widely applied as a pretreatment for the recovery of valuable metabolites, including polyphenols, carotenoids, and proteins [18,19]. Moreover, PEF-assisted aqueous extraction has been associated with reduced processing temperatures, lower solvent requirements, and improved extraction efficiency [17]. Consequently, PEF is regarded as a sustainable extraction strategy that enhances process performance while lowering energy consumption and protecting thermolabile compounds [20,21]. Owing to these advantages, PEF represents a rapid, environmentally friendly, and efficient technique for recovering bioactive constituents from plant materials. *C. sativa* leaves constitute a promising source of valuable phytochemicals with potential applications in both the food and pharmaceutical sectors.

Atmospheric Room Temperature Plasma (ARTP) has also gained considerable interest as a novel non-thermal pretreatment capable of improving the extraction of bioactive compounds from plant material. ARTP and other cold plasma technologies produce reactive nitrogen species (RNS) under ambient conditions, promoting surface micro-etching, partial disruption of cell wall structures, enhanced membrane permeability, and ultimately facilitating the release of cellular metabolites [22,23]. Several recent investigations have proven that plasma pretreatment can increase the extraction efficiency of phenolic compounds, antioxidant constituents, and other bioactives from various plant materials, including *Moringa oleifera* leaves, peach peels, laurel, spent coffee grounds, and edamame [24,25,26,27,28]. Furthermore, the use of nitrogen gas in the process minimizes degradation due to oxidation, making ARTP notably suitable for preserving heat-labile compounds such as terpenoids. However, no study has evaluated the synergistic combination of ARTP pretreatment with PEF-assisted extraction in hemp leaves, despite the complementary mechanisms of plasma-induced surface activation and electroporation-driven mass transfer.

Therefore, the current study aimed to improve the recovery of bioactive compounds from *C. sativa* leaves by combining ARTP pretreatment with PEF-assisted extraction. Response Surface Methodology (RSM) was employed to evaluate the effects of the distance between the plasma source and the sample surface (*D*, mm), sample thickness (*T*, mm), plasma power (*P*, %), nitrogen flow rate (*F*, L/min), and treatment time (*t*, min). Following ARTP pretreatment, all samples were subjected to PEF extraction under standardized operating conditions. Extraction performance was assessed by determining the total polyphenol content using the Folin–Ciocalteu assay, whereas antioxidant activity was evaluated using the FRAP and DPPH assays. Individual polyphenols for both the control and the optimal sample were determined by HPLC. The extract obtained under the predicted best-performing conditions was subsequently incorporated into vinegar as a model food system and further evaluated in a stability study considering extract concentration, storage temperature, and light exposure through RSM over a storage period of 28 days. This integrated experimental framework allowed the combined ARTP–PEF process to be evaluated not only in terms of extraction performance but also with respect to the compositional characteristics of the resulting extracts and their stability in an acidic food matrix.

## 2. Results and Discussion

### 2.1. Effect of ARTP Pretreatment on Antioxidant Responses

**Figure 1:**
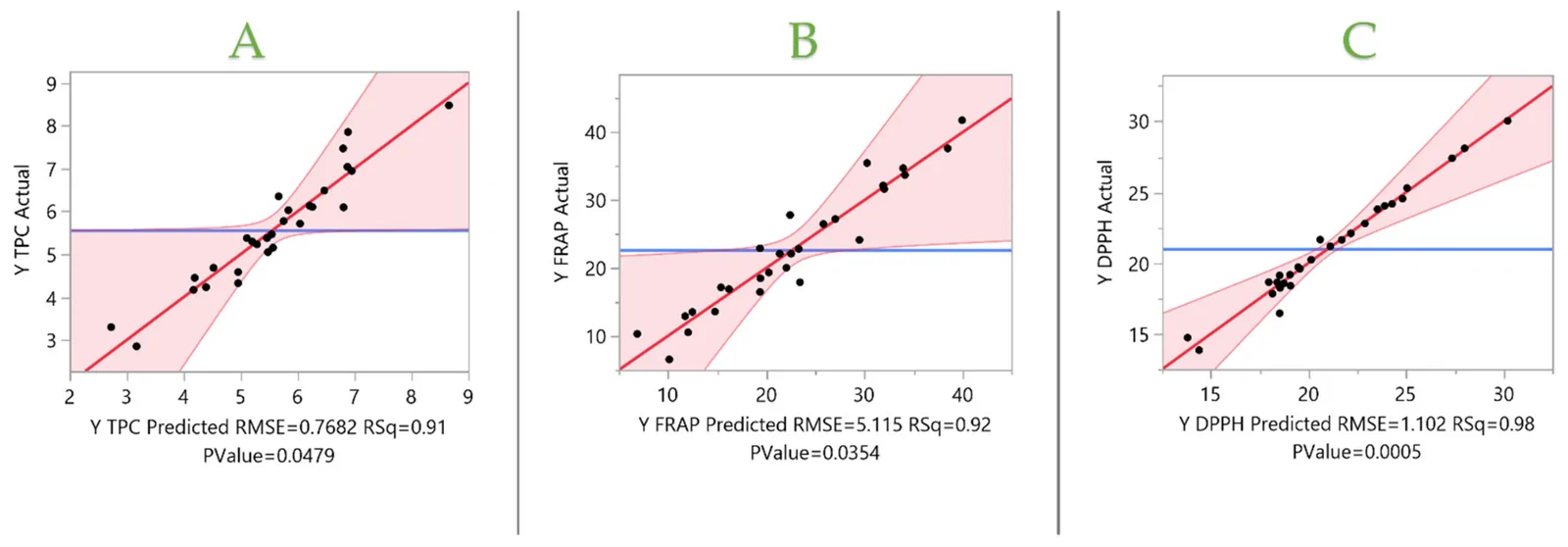
Actual vs. predicted values for the fitted response surface models of (**A**) total phenolic content (TPC), (**B**) ferric-reducing antioxidant power (FRAP), and (**C**) DPPH^•^ radical scavenging activity. The regression line (red) and 95% confidence band illustrate the correspondence between experimental and fitted values (R^2^ = 0.91, 0.92 and 0.98, respectively), noting that predictive performance—especially for TPC and FRAP—should be interpreted with caution. The red shaded band represents the 95% confidence interval of the regression fit, and the blue line denotes the 1:1 reference line.

The ARTP pretreatment produced substantial variability in the antioxidant responses of *Cannabis sativa* leaf extracts, confirming the sensitivity of the matrix to plasma-induced surface activation. Across the 28 CCD runs in Table 1, TPC ranged from 2.85 to 8.47 mg GAE/g dw, FRAP from 6.53 to 41.78 µmol AAE/g dw, and DPPH from 13.85 to 30.05 µmol AAE/g dw, consistent with the broad design space defined for the five ARTP factors. As shown in Figure 1, the fitted models reproduced the general trends of the experimental values, although their predictive performance should be interpreted with caution, particularly for TPC and FRAP.

#### 2.1.1. Total Polyphenol Content (TPC) Model

The TPC response was significantly influenced by quadratic effects of distance (X1^2^, *p* = 0.0046) and power (X3^2^, *p* = 0.0063), as well as the linear effect of nitrogen flow (X4, *p* = 0.0115). A weaker but notable contribution was observed for the quadratic term of nitrogen flow (X4^2^, *p* = 0.0409). These results (Table 2) indicate that TPC follows a curved response surface with clear optima at intermediate electrode–sample distances and plasma intensities, while nitrogen flow modulates the extent of phenolic release. The lack-of-fit test was non-significant (*p* = 0.168), confirming that the second-order model adequately captured the experimental variability.

#### 2.1.2. Ferric Reducing Antioxidant Power (FRAP) Model

FRAP was primarily driven by nitrogen flow (X4, *p* = 0.00076), followed by significant quadratic contributions of distance (X1^2^, *p* = 0.0126) and power (X3^2^, *p* = 0.0147), as presented in Table 2. The interaction X2 × X4 (*p* = 0.0270) also contributed, suggesting that bed thickness modulates the effect of gas flow on redox-active compounds. The model showed no evidence of lack-of-fit (*p* = 0.585), and the maximum achievable R^2^ was high (0.9907), indicating good model adequacy.

#### 2.1.3. 1,1-Diphenyl-2-picrylhydrazyl (DPPH^•^) Model

The DPPH^•^ response exhibited the richest structure, with strong contributions from both quadratic and interaction terms (Table 2). The most influential factor was nitrogen flow squared (X4^2^, *p* = 0.00002), followed by the linear effect of nitrogen flow (X4, *p* = 0.00006) and the quadratic effect of distance (X1^2^, *p* = 0.00041). Significant interactions included X2 × X4 (*p* = 0.0032), X2 × X5 (*p* = 0.0040), and X3 × X4 (*p* = 0.0121), indicating that plasma-induced radical chemistry depends strongly on the interplay between gas flow, material thickness, and treatment duration. The lack-of-fit test was non-significant (*p* = 0.918), confirming excellent model fit.

**Figure 2:**
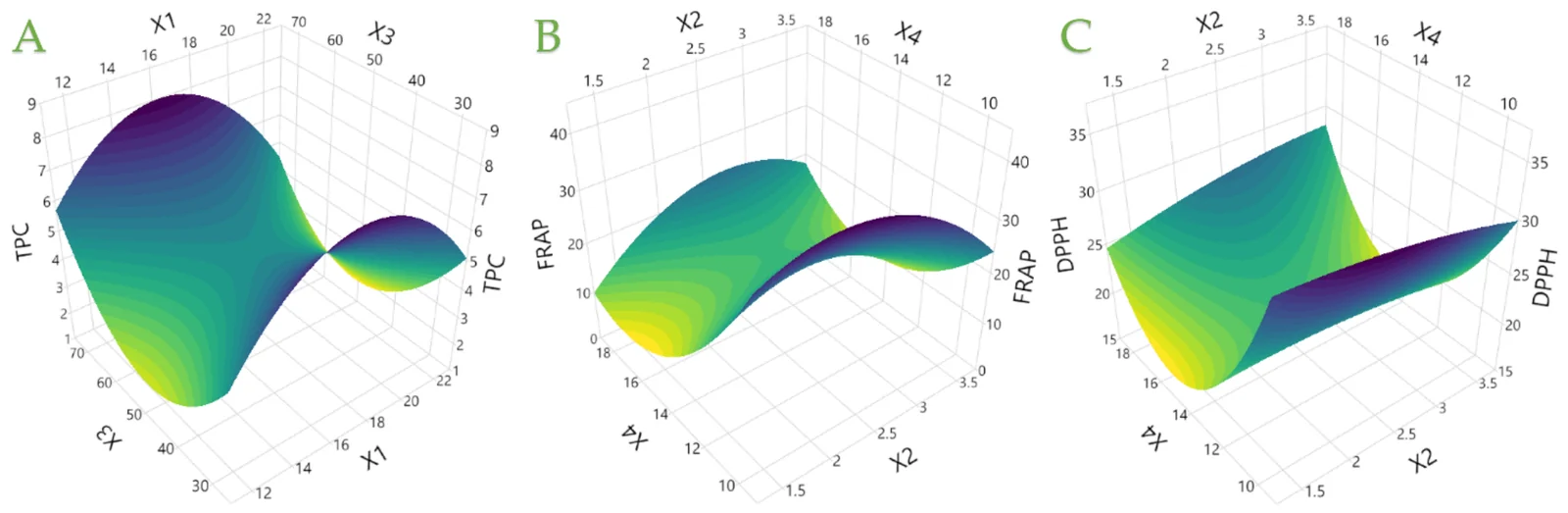
Response surface plots showing the interactive effects of ARTP processing variables on (**A**) total phenolic content (TPC) as a function of distance (X1) and power (X3), (**B**) ferric-reducing antioxidant power (FRAP) as a function of thickness (X2) and nitrogen flow (X4), and (**C**) DPPH^•^ radical scavenging activity as a function of thickness (X2) and nitrogen flow (X4). All surfaces exhibit clear curvature, consistent with the significant quadratic and interaction terms identified in the ANOVA.

The response surface plots (Figure 2) illustrate the non-linear behavior of all three antioxidant responses. TPC exhibited a curved surface with a maximum at intermediate electrode–sample distances and high plasma power (Figure 2A), consistent with the significant quadratic terms X1^2^ and X3^2^. FRAP increased sharply with nitrogen flow, while sample thickness modulated this effect (Figure 2B), in agreement with the X2 × X4 interaction. DPPH^•^ showed the strongest curvature, driven primarily by nitrogen flow (X4 and X4^2^), with thickness shifting the location of the optimum (Figure 2C). Model adequacy statistics for all three antioxidant responses are summarized in Table 3, indicating acceptable correspondence between fitted and experimental values and no significant lack-of-fit across the RSM models.

Distance between the sample and the ARTP source, sample thickness, ARTP power, nitrogen flow rate, and treatment duration interactively influence molecular responses such as total phenolic content (TPC), ferric-reducing antioxidant power (FRAP), and DPPH radical scavenging activity. These parameters collectively determine the generation, diffusion, and reactivity of reactive oxygen and nitrogen species (RONS), which drive oxidative and structural modifications in plant tissues.

Shorter plasma–sample distances increase RONS density and energy flux, enhancing cell wall disruption and facilitating the release of phenolic compounds. However, excessive proximity may lead to over-oxidation and degradation of thermo-sensitive molecules. Intermediate distances (≈15–20 mm) provide a balance between activation and preservation, consistent with previous observations in plasma-treated plant materials [25,28].

Sample thickness strongly influences RONS penetration depth. Thin layers (≈1 mm) expose a larger surface area, enabling uniform oxidative activation and higher extraction yields. In contrast, thicker layers (>3 mm) hinder RONS diffusion, resulting in lower FRAP and DPPH values [29]. This trend aligns with the curvature observed in plots B and C, where increased thickness reduces plasma effectiveness.

Plasma power determines electron density and the rate of RONS generation. Moderate power levels (≈15 V, 50 kHz) promote controlled oxidation and surface activation without causing thermal degradation. Higher power intensities increase RONS concentration but may induce excessive oxidation, molecular fragmentation, and loss of antioxidant capacity [30,31].

Nitrogen flow rate regulates the composition and stability of reactive nitrogen species (NO, NO_2_^−^, N_2_^+^) relative to oxygen species. Intermediate flow rates maintain a balanced oxidative environment, enhancing FRAP values. Excessive flow reduces RONS residence time and limits their interaction with molecular targets, thereby diminishing plasma efficiency [32,33].

Treatment duration defines the cumulative exposure of the sample to RONS. In dense plant matrices such as spent coffee grounds, longer plasma exposure (>10 min) is often required to achieve sufficient cell wall disruption [28]. However, in the present study, cannabis leaves exhibited optimal molecular responses at very short ARTP duration (1 min). The same optimal duration for plasma treatment was shown for peach peel powder [25]. Cannabis leaves possess a thin laminar structure and high surface porosity, allowing rapid RONS penetration and immediate surface activation. Longer exposure times (>5–10 min) resulted in decreased TPC, FRAP, and DPPH^•^ values, indicating oxidative degradation of phenolics rather than further enhancement. This highlights the matrix-dependent nature of ARTP efficiency and the importance of tailoring plasma parameters to specific plant tissues.

**Figure 3:**
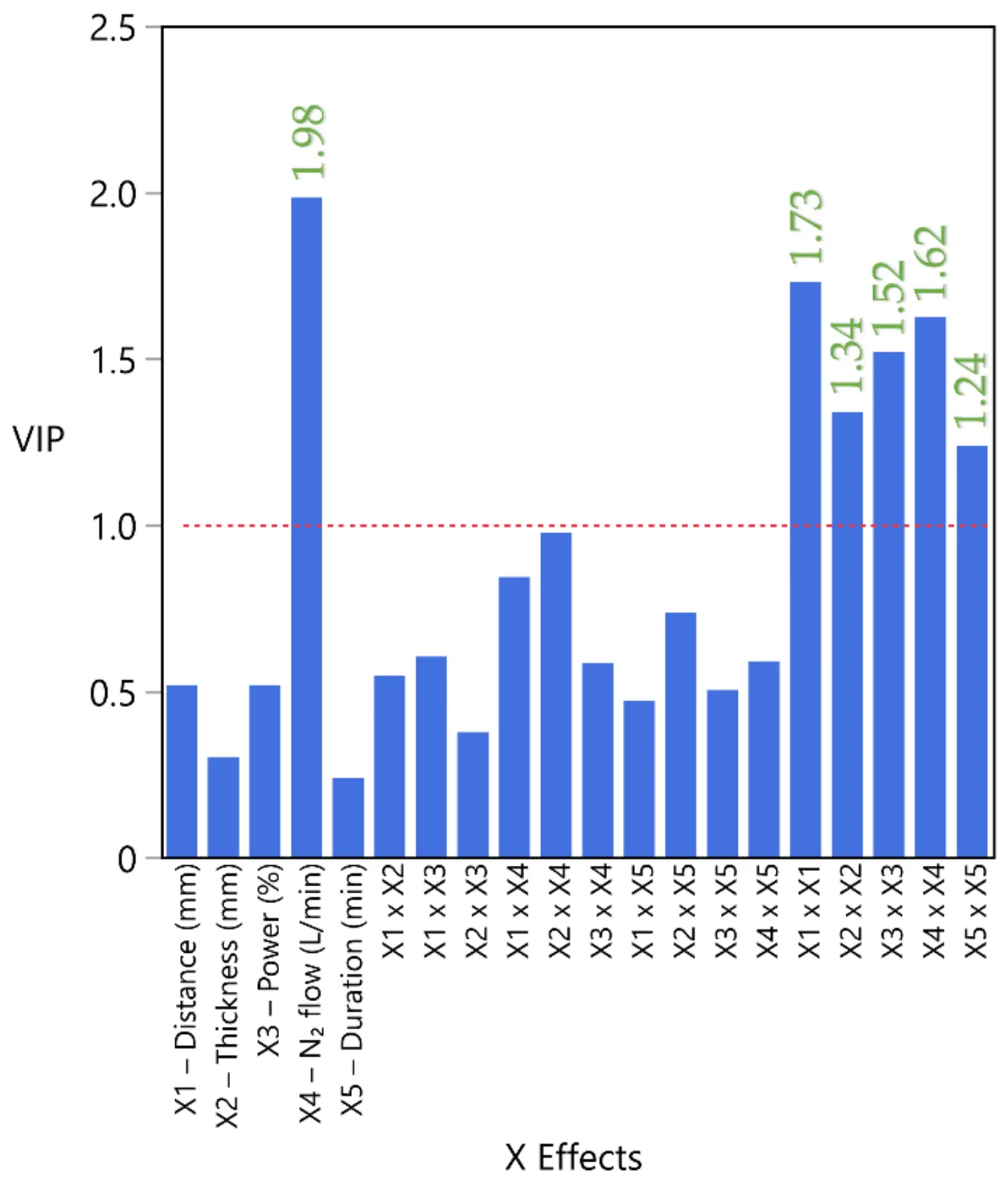
Variable Importance in Projection (VIP) scores from the PLS model, indicating the relative contribution of each main effect and interaction term to the fitted model. The red dashed line marks the VIP threshold of 1.0.

The PLS-VIP analysis (Figure 3) indicated that the VIP analysis showed that the quadratic terms of nitrogen flow (X4^2^), distance (X1^2^), power (X3^2^), thickness (X2^2^), and treatment duration (X5^2^) were the most influential terms (VIP > 1.2), indicating that nonlinear effects were the predominant contributors to the multivariate antioxidant response. Among the linear factors, nitrogen flow (X4) showed the highest VIP value (1.98), identifying it as the most influential individual processing factor.

In contrast, the interaction terms generally exhibited lower VIP values (<1), with X2 × X4 (thickness × nitrogen flow) being the only interaction approaching the conventional importance threshold (VIP = 0.98). This suggests that the antioxidant responses were governed predominantly by nonlinear individual-factor effects, while synergistic contributions between pairs of factors were comparatively limited. These observations are consistent with the RSM analysis, in which significant quadratic effects were observed for the processing variables.

**Figure 4:**
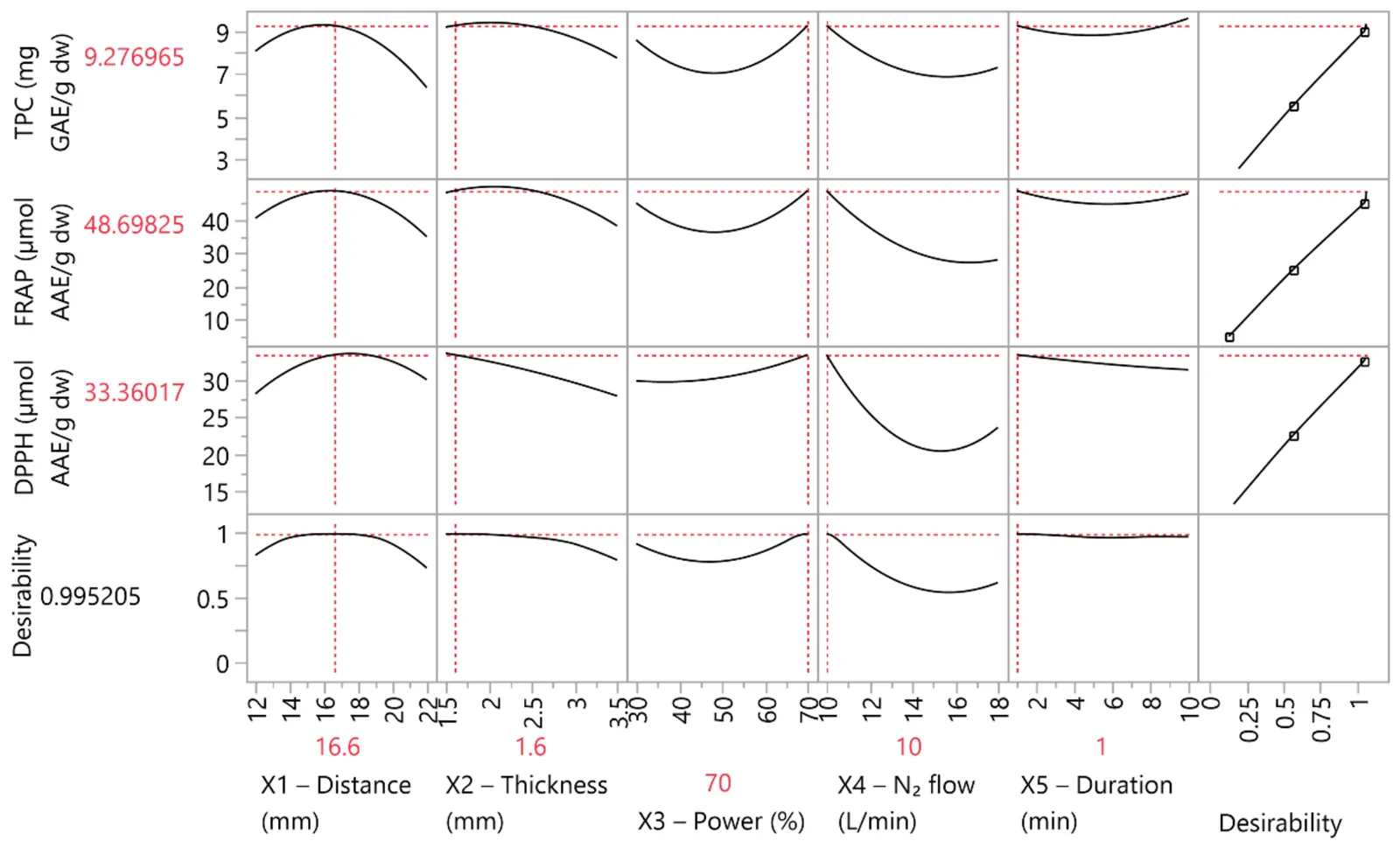
PLS prediction profiler illustrating the effect of the five ARTP processing variables on TPC, FRAP, DPPH and overall desirability.

The PLS prediction profiler (Figure 4) demonstrated clear nonlinear effects for all responses, with strong curvature in X1, X3 and X4, consistent with the significant quadratic terms identified in the ANOVA and VIP analysis. The optimum was achieved at X1 = 16.6 mm, X2 = 1.6 mm, X3 = 70%, X4 = 10 L/min and X5 = 1 min, resulting in predicted values of 9.28 mg GAE/g dw (TPC), 48.70 µmol AAE/g dw (FRAP) and 33.36 µmol AAE/g dw (DPPH), with an overall desirability of 0.995.

The response surface models developed in this study should be interpreted with appropriate caution. Although the fitted models exhibited relatively high R^2^ values, the substantially lower adjusted R^2^ values for TPC (0.65) and FRAP (0.68) indicate that part of the apparent explained variance may be attributable to model complexity and limited residual degrees of freedom rather than strong predictive capability. The experimental design was a face-centered central composite design, which, with 28 runs and 21 model terms, leaves few degrees of freedom for estimating pure error; consequently, the lack-of-fit tests have limited statistical power, and non-significant lack-of-fit should not be interpreted as definitive evidence of model adequacy. Furthermore, the predicted optimum lies at the boundary of the investigated factor space, meaning that the identified settings represent the best-performing conditions within the experimental range rather than a true stationary point of the response surface. This is consistent with the validation results, where deviations of approximately 19% for TPC and FRAP indicate moderate predictive uncertainty. Taken together, these considerations suggest that the RSM models are suitable for describing general trends and identifying promising operating regions, but they should not be regarded as fully robust predictive tools without further refinement or expansion of the experimental domain.

### 2.2. Validation of the Optimized ARTP Conditions

The best conditions within the investigated range for ARTP predicted by the RSM model (16.6 mm electrode–sample distance, 1.6 mm sample thickness, 70% plasma intensity, 10 L min^−1^ nitrogen flow rate, and 1 min treatment time) were experimentally validated, and the results are presented in Table 4. Although the experimental values were lower than those predicted by the model, the deviations remained below 20% for all responses, indicating acceptable agreement between predicted and experimental values. The deviations were −18.9% for TPC, −19.5% for FRAP, and only −4.6% for DPPH^•^, suggesting comparatively better predictive agreement for DPPH^•^. These differences are likely attributable to the number of terms included in the second-order models and the limited experimental degrees of freedom, or minor experimental fluctuations during plasma treatment and extraction rather than model inadequacy, as evidenced by the non-significant lack-of-fit for all responses (Table 3).

The deviations between predicted and experimental values for TPC and FRAP were approximately 19%. Such differences should be considered when assessing the practical applicability of the model, as prediction errors may arise from biological and analytical variability and from the approximation inherent to response surface models. Since no universal acceptance criterion was established for the present extraction system, the validated condition should be regarded as a promising operating point within the investigated experimental domain rather than as a definitive industrial optimum. Further validation at pilot scale would be required prior to industrial implementation.

Comparison with the control samples demonstrated the effectiveness of ARTP pretreatment prior to PEF extraction. Under the best conditions within the investigated range, TPC increased by 23.0%, while antioxidant capacity exhibited even greater improvements, with FRAP and DPPH values increasing by 68.6% and 46.1%, respectively, compared with PEF extraction alone. These findings indicate that ARTP pretreatment not only enhanced the extraction yield of phenolic compounds but also promoted the recovery of compounds with high antioxidant potential.

Interestingly, the increase in antioxidant capacity was proportionally greater than the increase in TPC, particularly for FRAP. This observation suggests that ARTP treatment may preferentially enhance the extraction or availability of highly active phenolic constituents rather than simply increasing the total amount of phenolics. The enhanced extraction observed following ARTP pretreatment may be associated with multiple physicochemical effects of atmospheric plasma. Reactive oxygen and nitrogen species generated in the plasma environment may interact with plant-cell surfaces and promote oxidation or modification of cell wall components, potentially increasing tissue permeability. UV photons and other energetic plasma species may further contribute to localized structural modifications. Such effects could facilitate subsequent PEF treatment by increasing the accessibility of intracellular compounds and reducing mass-transfer limitations [30,34,35,36,37]. However, these mechanisms are proposed based on previously reported plasma–plant interactions and were not directly characterized in the present study. This hypothesis is further supported by the HPLC–DAD analysis (Section 3.3), which confirmed changes in the concentration of individual phenolic compounds following ARTP pretreatment.

Finally, the combined ARTP–PEF treatment also outperformed the untreated control (no plasma–no PEF), confirming that both technologies contributed to improved extraction efficiency. However, the substantially higher values obtained with ARTP followed by PEF compared with PEF alone demonstrate that plasma pretreatment provided an additional synergistic effect, reinforcing the potential of integrating these two non-thermal technologies for the recovery of antioxidant-rich extracts from *Cannabis sativa* leaves.

The present study expands current knowledge on non-thermal extraction technologies by demonstrating, that atmospheric room-temperature plasma (ARTP) can effectively enhance the efficiency of subsequent PEF-assisted extraction of antioxidant compounds from *Cannabis sativa* leaves. Although studies combining ARTP and PEF for hemp extraction are currently unavailable, recent investigations on other plant matrices support the proposed mechanism. Kyriakoudi et al. [28] reported that cold atmospheric plasma (CAP) pretreatment prior to ultrasound-assisted extraction increased total phenolic content by approximately 31% (from 19.0 to 24.9 mg GAE/100 g dry weight) and chlorogenic acid recovery by nearly 40% (from 79.7 to 111.3 mg/100 g dry weight) from spent coffee grounds, demonstrating that plasma-induced surface modification substantially improves solvent accessibility and mass transfer. Similarly, CAP pretreatment combined with ultrasound-assisted extraction significantly enhanced both phenolic recovery and antioxidant activity in cornelian cherry pomace compared with ultrasound extraction alone, confirming the synergistic potential of sequential green technologies [38].

The nonlinear influence of plasma operating parameters noticed in the current study is also consistent with previous reports showing that plasma efficiency depends on carefully optimized treatment conditions rather than maximum energy input. Moderate plasma exposure has been associated with partial disruption of cell wall structures, enhanced tissue permeability and ameliorated diffusion of cellular metabolites, whereas excessive treatment may promote oxidation of susceptible phytochemicals, limiting extraction efficiency. These observations support the hypothesis that ARTP pretreatment enhanced the effectiveness of the subsequent PEF process by facilitating solvent penetration and electroporation-assisted release of intracellular antioxidants [28,30,37,39]. Regarding the present study, cell wall modification and increased tissue permeability are proposed as a mechanism rather than as a directly demonstrated structural effect, since no SEM application was included in the experimental design. Compared with other green extraction approaches applied to *Cannabis sativa*, the proposed ARTP–PEF strategy represents an additional process intensification step. Lazarević et al. [40] reported that ultrasound-assisted extraction produced higher extraction yield (17.54% vs. 15.28%), total phenolic content (1.78 vs. 1.05 mg GAE mL^−1^), total flavonoid content (0.67 vs. 0.36 mg CE mL^−1^) and cannabidiol concentration (0.88 vs. 0.43 mg mL^−1^) than conventional extraction, emphasizing the advantages of intensified non-thermal extraction processes. Likewise, recent comparative studies have demonstrated that extraction technology strongly influences both the phytochemical composition and antioxidant capacity of hemp extracts [41]. In agreement with these findings, the improved antioxidant recovery achieved in the present study suggests that ARTP pretreatment complements PEF through distinct yet synergistic mechanisms, providing a sustainable strategy for maximizing the recovery of bioactive compounds from hemp leaves while preserving their functional properties.

### 2.3. HPLC Analysis

**Figure 5:**
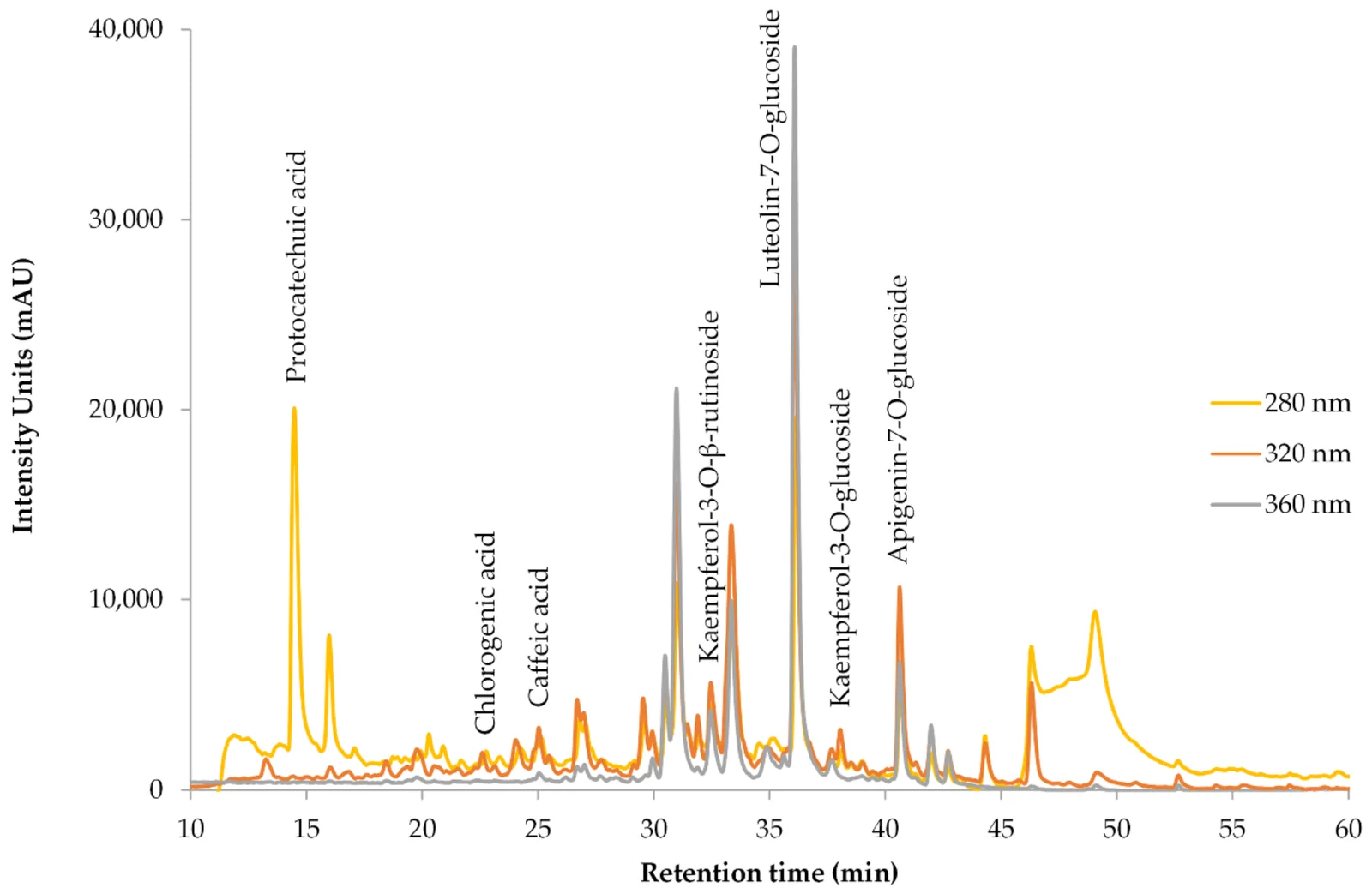
HPLC chromatogram of phenolic compounds obtained under Optimal ARTP treatment at 280, 320, and 360 nm.

To determine whether the enhanced antioxidant activity observed after ARTP pretreatment was associated with compositional changes in the extracted phytochemical profile, the optimized extracts were further characterized by HPLC–DAD. According to the chromatogram obtained under the optimal ARTP conditions (Figure 5), protocatechuic acid and luteolin-7-*O*-glucoside were the compounds identified and quantified above the limit of quantification (LOQ) under the applied analytical conditions, whereas other investigated compounds were detected below the LOQ or limit of detection (LOD).

Quantitative results (Table 5) revealed that ARTP pretreatment significantly increased the concentration of the two key phenolic compounds identified compared with PEF extraction alone. Protocatechuic acid increased from 0.44 ± 0.02 mg/g to 0.54 ± 0.03 mg/g, while luteolin-7-*O*-glucoside increased from 0.45 ± 0.02 mg/g to 0.51 ± 0.03 mg/g (*p* < 0.05). The untreated control (No Plasma–No PEF) consistently exhibited the lowest concentrations (0.31–0.32 mg/g), confirming the contribution of both ARTP and PEF to phenolic release. All other detected compounds remained below the limit of quantification, indicating that no additional phenolic compounds were reliably quantified under the applied HPLC-DAD conditions. Analytical performance parameters for all investigated compounds—including calibration equations, coefficients of determination (R^2^), retention times, UV maxima, and LOD/LOQ values—are provided in Table A1, while recovery and precision data for the HPLC-DAD method are summarized in Table A2.

These findings are consistent with the increases observed in TPC, FRAP, and DPPH values, suggesting that the improved antioxidant capacity of the ARTP-treated extracts is driven not only by higher total phenolic levels but also by the elevated recovery of individual bioactive molecules. The selective enhancement of protocatechuic acid and luteolin-7-*O*-glucoside—both compounds with well-documented antioxidant properties—supports the hypothesis that ARTP facilitates cell wall disruption and promotes the release of bound or glycosylated phenolics. Overall, the HPLC results corroborate the RSM-based optimization outcomes and demonstrate that ARTP pretreatment can effectively intensify the extraction of structurally relevant phenolics from *Cannabis sativa* leaves.

However, the interpretation of the phenolic profile should be limited to the compounds reliably quantified under the applied HPLC–DAD conditions. Only protocatechuic acid and luteolin-7-*O*-glucoside were detected above the limit of quantification, whereas several other target phenolics were present below the LOQ or LOD and therefore could not be quantified with sufficient analytical confidence. Although the chromatograms displayed additional peaks of notable intensity, these correspond to compounds for which authentic standards were not available, preventing definitive identification or quantification. As a result, the present analysis cannot support broad conclusions regarding the overall phenolic composition of the extracts. Furthermore, the diode-array detector alone cannot exclude the possibility of plasma-induced formation or transformation of phenolic derivatives, and the absence of newly quantified compounds should not be interpreted as evidence that no structural modifications occurred. Future work incorporating complementary chromatographic and mass-spectrometric techniques, as well as targeted optimization for higher-molecular-weight flavonoids, will be necessary to more comprehensively characterize the phenolic profile of ARTP-treated hemp extracts.

### 2.4. Vinegar Stability Study

The objective of the stability study was to evaluate the stability of the optimized ARTP–PEF extract when incorporated into an acidic model matrix under different extract concentrations, temperatures and light conditions. The factorial ANOVA revealed that extract concentration (X1) was by far the dominant factor affecting all antioxidant responses (TPC, FRAP, DPPH) across all storage days (*p* < 0.001). The experimental design and initial antioxidant values (Day 0) for all treatments are summarized in Table 6, providing the baseline for evaluating temporal stability across the 28-day storage period. Temperature (X2) and light exposure (X3) showed no significant main effects on TPC and only marginal effects on FRAP and DPPH at specific time points. Interaction effects were generally weak, with the exception of X1 × X2 and X1 × X3 at Day 14 for FRAP (*p* < 0.01), indicating that mid-storage conditions modulated antioxidant stability. The statistically significant model terms for each antioxidant response across all storage days are summarized in Table 7. All models exhibited high R^2^ values (0.97–0.999) with no evidence of lack-of-fit. Model adequacy statistics for all fitted stability models are presented in Table 8, confirming their adequacy for describing stability trends across storage days.

Overall, cannabis vinegar exhibited good antioxidant stability over 28 days, particularly in samples enriched with extract (X1 = 1.2 g/L). A consolidated summary of stability trends and factor effects is provided in Table 9. TPC showed minimal degradation, whereas FRAP declined progressively and DPPH^•^ followed a characteristic activation–peak–decay profile. Temperature was the main destabilizing factor, while light had a minor effect. The most stable formulation was obtained at high extract concentration, low temperature, and dark storage.

**Figure 6:**
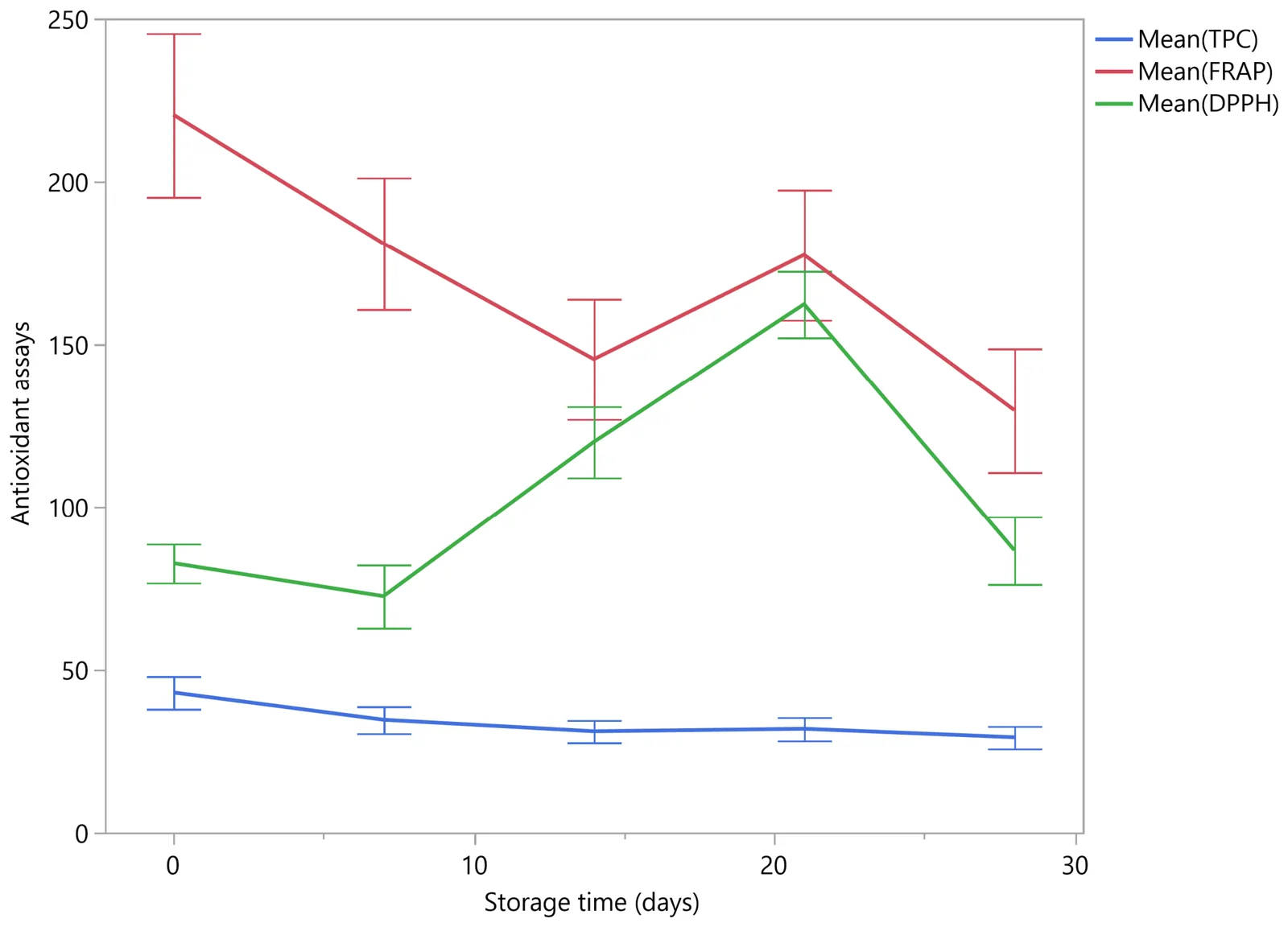
Stability of antioxidant assays (TPC, FRAP, DPPH) in cannabis vinegar during 28-day storage. TPC in mg GAE/L; FRAP and DPPH^•^ in μmol AAE/L.

The temporal evolution of antioxidant stability across all treatments is illustrated in Figure 6, showing distinct degradation patterns for TPC, FRAP, and DPPH over the 28-day storage period. The increase in DPPH activity during intermediate storage may reflect changes in the chemical composition or antioxidant reactivity of phenolic constituents, potentially including hydrolysis or transformation of conjugated compounds; however, these mechanisms were not directly investigated in the present study and are therefore presented only as hypotheses. TPC, FRAP and DPPH measure different chemical properties and may consequently exhibit different temporal profiles during storage.

To more accurately evaluate antioxidant stability during storage, all responses in the vinegar experiment were expressed as retention relative to each sample’s own Day 0 value, rather than as absolute concentrations. This approach distinguishes the effect of extract concentration on initial antioxidant levels from its effect on temporal preservation. Percentage retention was calculated as Retention (%) = (*C*_t_/*C*_0_) × 100, where *C*_t_ represents the measured value at each storage day and *C*_0_ the corresponding baseline value for the same treatment. When expressed in this form, TPC exhibited relatively high stability across all conditions, with most treatments retaining approximately 65–80% of their initial values after 28 days. FRAP showed a more pronounced decline, particularly at 40 °C, where retention typically decreased to 55–70%, indicating temperature-driven degradation of redox-active constituents. DPPH displayed a characteristic polyphasic pattern, with retention increasing during intermediate storage (Days 14–21) before declining at Day 28. This non-monotonic behavior may reflect matrix-dependent transformations such as acid-catalyzed hydrolysis or changes in the reactivity of conjugated phenolics; however, these mechanisms were not directly investigated and are presented only as hypotheses. Overall, expressing antioxidant responses as retention clarified that extract concentration primarily determines initial antioxidant levels, whereas temperature is the dominant factor governing degradation kinetics during storage.

**Figure 7:**
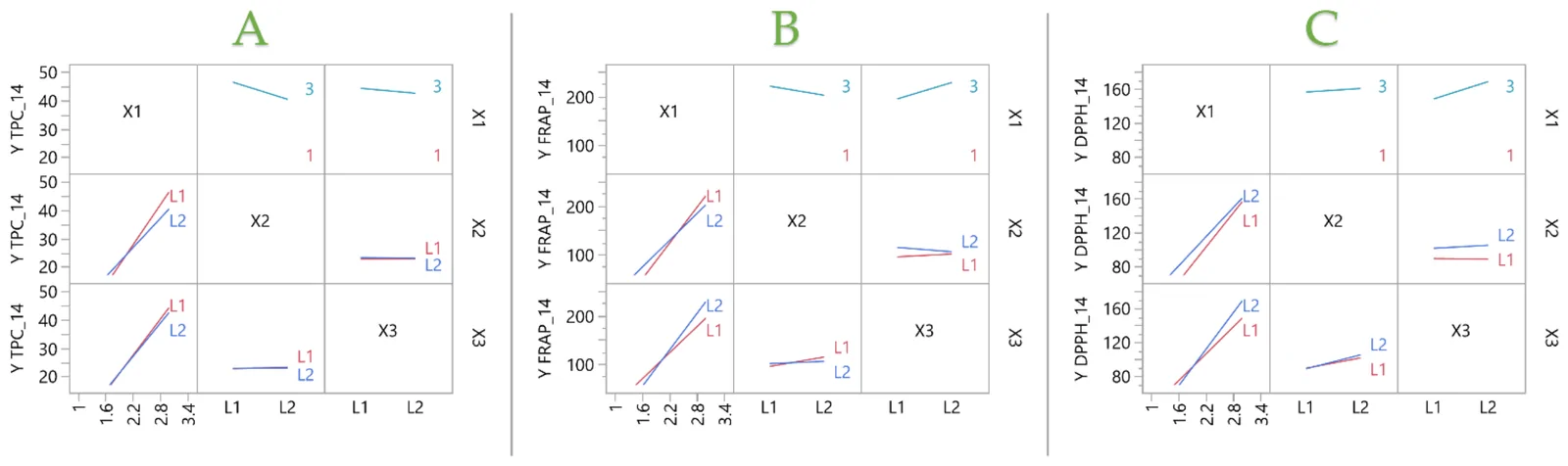
Interaction profiler plots for (**A**) TPC, (**B**) FRAP and (**C**) DPPH^•^, illustrating the combined effects of extract concentration (X1), temperature (X2) and light exposure (X3) on the antioxidant responses at Day 14.

Figure 7 illustrates the interaction profiler plots for Day 14, which was the time point with the strongest interaction effects. For TPC, a moderate X1 × X2 interaction was observed, indicating that temperature modulated the effect of extract concentration. FRAP exhibited pronounced interactions (X1 × X2, X1 × X3, X2 × X3), confirming that antioxidant stability at mid-storage was influenced by multiple factor combinations. DPPH showed a dominant main effect of X1 with weaker interaction patterns.

To the best of our knowledge, no previous study has evaluated the incorporation of hemp leaf phenolic extracts into vinegar as a functional food matrix. Most food applications reported to date have focused on fermented dairy products [42], meat products [43], bakery formulations or hemp seed-derived ingredients [44] rather than extracts obtained from hemp leaves. The successful enrichment of vinegar achieved in the present study therefore broadens the potential food applications of hemp-derived bioactive compounds and demonstrates the feasibility of using acidic food matrices as carriers of natural antioxidants.

## 3. Materials and Methods

### 3.1. Chemicals and Reagents

Ethanol, acetic acid, gallic acid, and the Folin–Ciocalteu reagent were bought from Panreac Co. (Barcelona, Spain). Hydrochloric acid, methanol, 2,2-diphenyl-1-picrylhydrazyl (DPPH), and 2,4,6-tris(2-pyridyl)-s-triazine (TPTZ) were obtained from Sigma-Aldrich (Darmstadt, Germany). Anhydrous sodium carbonate was purchased from Penta (Prague, Czech Republic). Iron (III) chloride was purchased from Merck (Darmstadt, Germany). For the polyphenol analysis by HPLC, high purity standards (≥99% *w*/*w*) were used, which were obtained from MetaSci (Toronto, ON, Canada). Deionized water was used throughout all experiments.

### 3.2. Leaf Material

Leaves of industrial hemp (*Cannabis sativa* var. Futura 75), excluding the flowering and fruiting tops, were supplied by CBD Extraction I.K.E. (Farsala, Greece). The plant material originated from cultivation fields located in the Farsala area (39°17′47″ N, 22°22′51″ E; coordinates obtained from Google Earth version 10.110.84.3). Upon collection, the leaves were thoroughly washed with distilled water to remove impurities and gently dried using absorbent paper towels. Subsequently, the samples were freeze-dried in a Biobase BK-FD10P lyophilizer (Jinan, China). The dehydrated leaves were milled with an electric grinder, and the resulting powder was sieved using an Analysette 3 PRO sieve shaker (Fritsch GmbH, Idar-Oberstein, Germany). Only the fraction with particle size < 400 μm was used in all experiments. The final powdered material was stored in airtight containers at −40 °C until further analysis.

### 3.3. Experimental Design and Optimization Workflow

#### 3.3.1. ARTP Pretreatment of Cannabis Powder

A structured experimental design was implemented to investigate the effect of Atmospheric Room Temperature Plasma (ARTP) pretreatment on the extractability and antioxidant stability of *Cannabis sativa* leaf polyphenols. Plasma treatments were performed using a Piezobrush^®^ PZ3-i device (Relyon Plasma GmbH, Regensburg, Germany) operating under Piezoelectric Direct Discharge (PDD^®^) technology with high-purity nitrogen (N_2_) as the working gas. Prior to experimentation, the plasma device was inspected to ensure stable discharge behavior and consistent operating conditions across all treatments. Freeze-dried cannabis leaf powder (<400 μm) was evenly distributed in polystyrene culture dishes (90 mm) to form a uniform bed layer. Because plasma irradiation can produce spatially heterogeneous surface effects, the irradiated plant powder was subjected to post-treatment homogenization to eliminate spatial variance and sampling bias. To strictly prevent particle size reduction and alterations to the specific surface area—which would act as confounding factors in the subsequent extraction—the plasma-treated powder passed three consecutive times through a 400 µm mesh sieve. This sieving process disrupted electrostatic agglomerates formed during plasma exposure, ensuring a homogeneous and representative dry matrix for the extraction trials. All treatments were conducted under ambient laboratory conditions inside a ventilated fume hood.

Five independent variables were selected based on prior ARTP studies on botanical matrices, with the design space intentionally restricted to avoid over-oxidation of sensitive flavonoids and phenolic acids. The factors and their coded levels are shown in Table 10.

In this study, plasma power was expressed as a percentage because the Piezobrush^®^ PZ3-i generator provides a relative output control rather than an absolute wattage readout. The percentage corresponds to the internal duty-cycle setting of the device, which regulates the discharge intensity but is not directly convertible to W or V·A.

These ranges were selected to ensure mild surface activation without inducing thermal or radical-driven degradation of cannabis polyphenols.

A five-factor, three-level Central Composite Design (CCD) was employed, consisting of 28 experimental runs including center points for reproducibility. Design evaluation indicated adequate statistical power for linear and interaction terms (>0.92) and acceptable precision for estimating quadratic effects. Each ARTP-treated sample was subsequently subjected to extraction under fixed, previously optimized PEF conditions (Section 3.3.2). Responses included total polyphenol content (TPC), ferric-reducing antioxidant power (FRAP), DPPH radical scavenging activity, and quantification of individual polyphenols by HPLC-DAD.

The CCD enabled the development of second-order polynomial models describing the individual and interactive effects of ARTP parameters on extractability and antioxidant indices, supporting multi-response optimization.

#### 3.3.2. PEF Extraction Under Fixed Optimal Conditions

To isolate the effect of ARTP pretreatment, all extractions were performed under the previously optimized PEF conditions established for *Cannabis sativa* var. Futura 75 leaves by our team [41]. The extraction medium consisted of 50% *v*/*v* aqueous ethanol at a liquid-to-solid ratio of 20 mL/g, and PEF treatment was applied using an electric field strength of 0.9 kV/cm, a pulse duration of 10 μs, a pulse period of 1000 μs, while the extraction time was 25 min. These conditions were previously shown to maximize TPC, FRAP, DPPH, and individual polyphenol yields. After PEF treatment, samples were centrifuged at 10,000× *g* for 10 min, and supernatants were collected for chemical analysis. This fixed-PEF approach allowed the ARTP pretreatment to be evaluated as an upstream intensification step without confounding interactions between plasma and electroporation parameters.

#### 3.3.3. Multi-Response Optimization of ARTP Conditions

Second-order polynomial models derived from the CCD were used to perform multi-response optimization through a desirability function approach. Individual desirability functions were defined for TPC, FRAP, DPPH, and the HPLC-identified polyphenols consistently detected above the LOQ (protocatechuic acid and luteolin-7-*O*-glucoside). A global desirability score was then computed to identify the optimal combination of ARTP parameters that simultaneously maximized all responses. The predicted optimal settings were subsequently selected for experimental validation.

#### 3.3.4. Validation of the Optimal ARTP Conditions

Three independent ARTP treatments were performed under the optimal conditions predicted by the desirability analysis. Extracts were prepared using the fixed PEF protocol described above. Experimental values for all responses were compared with model predictions to assess agreement between predicted and experimental values.

#### 3.3.5. Comparison with Control Extract

To isolate the specific contribution of ARTP pretreatment to the extraction efficiency of *Cannabis sativa* leaves, a control extract was prepared using the previously optimized PEF protocol without any ARTP pretreatment. This PEF-only extract served as the technological baseline for all comparative analyses.

Control samples were processed under identical conditions to the ARTP-treated samples, including solvent composition (50% *v*/*v* aqueous ethanol), liquid-to-solid ratio (20 mL/g), electric field strength (0.9 kV/cm), pulse duration (10 μs), pulse period (1000 μs), and extraction time (25 min). All downstream steps—centrifugation, supernatant collection, freeze-drying, and storage—were performed in parallel with the ARTP + PEF samples to ensure full methodological consistency. Comparative evaluation focused on total polyphenol content (TPC), ferric-reducing antioxidant power (FRAP), DPPH^•^ radical scavenging activity, and individual polyphenols quantified by HPLC-DAD. This comparison enabled quantification of ARTP-specific improvements in extractability and antioxidant capacity relative to the PEF-only baseline.

#### 3.3.6. Total Polyphenol Content (TPC)

The total polyphenol content (TPC) was quantified according to a previously reported procedure [45]. In brief, 0.10 mL of the extract was mixed with 0.10 mL of Folin–Ciocalteu reagent. After allowing the reaction to proceed for 2 min, 0.80 mL of a 5% (*w*/*v*) sodium carbonate solution was added. The reaction mixture was subsequently incubated at 40 °C for 20 min, after which its absorbance was measured at 740 nm using a Shimadzu UV-1700 PharmaSpec spectrophotometer (Kyoto, Japan). The concentration of total polyphenols (*C*_TP_) was estimated from a calibration curve prepared with gallic acid as the reference standard. Finally, the total polyphenol yield (*Y*_TP_) was expressed as milligrams of gallic acid equivalents (GAE) per gram of dry weight (dw).

#### 3.3.7. Ferric-Reducing Antioxidant Power (FRAP) Assay

The ferric-reducing antioxidant power (FRAP) of the extracts was determined following the method described by Shehata et al. [46]. Briefly, 0.05 mL of an appropriately diluted extract was combined with 0.05 mL of FeCl_3_ solution (4 mM prepared in 0.05 M HCl). After incubation at 37 °C for 30 min, 0.9 mL of TPTZ solution (1 mM in 0.05 M HCl) was added immediately, and the absorbance was recorded at 620 nm following an additional 5 min of reaction. Ferric-reducing capacity (*P*_R_) was quantified using an ascorbic acid calibration curve (*C*_AA_) prepared in 0.05 M HCl over a concentration range of 50–500 μM. The results were expressed as μmol ascorbic acid equivalents (AAE) per gram of dry weight (dw).

#### 3.3.8. DPPH^•^ Antiradical Activity Assay

The antiradical activity (*A*_AR_) of the polyphenol extracts obtained from the dried hemp material was determined using a slightly modified DPPH^•^ assay according to the procedure described by Shehata et al. [46]. Briefly, 4 mL of the extract was mixed with 1 mL of a 0.1 mM DPPH^•^ solution prepared in methanol. The reaction mixture was then incubated at room temperature in the absence of light for 30 min before measuring the absorbance at 515 nm. A blank solution, consisting of methanol and the DPPH^•^ reagent without the sample extract, was prepared and its absorbance was recorded immediately under the same experimental conditions.

The antiradical activity (*A*_AR_) was determined through an ascorbic acid calibration curve (*C*_AA_, 100–1000 μmol/L in methanol, R^2^ = 0.9926) according to the following Equation (1):

(1) $$A_{\mathrm{AR}}\;(\mu \mathrm{mol}\;\mathrm{AAE}/g\;dw)\;=\;\frac{C_{AA}\;\times \;V}{w}$$

where *V* represents (in L) the entire volume of the extraction medium and *w* (in g) represents the dried weight (dw) of the material. The results were expressed as μmol ascorbic acid equivalents (AAE) per g dw.

#### 3.3.9. HPLC Quantification of Polyphenolic Compounds

Individual polyphenolic compounds present in the extracts were identified and quantified by high-performance liquid chromatography (HPLC) following the analytical procedure previously reported by our team [41]. Chromatographic analysis was performed using a Shimadzu CBM-20A HPLC system coupled with a Shimadzu SPD-M20A diode array detector (DAD) (Shimadzu Europa GmbH, Duisburg, Germany). Separation was achieved on a Phenomenex Luna C18(2) column (100 Å, 5 μm, 4.6 mm × 250 mm; Phenomenex Inc., Torrance, CA, USA), maintained at 40 °C throughout the analysis.

The mobile phase consisted of 0.5% formic acid in water (solvent A) and 0.5% formic acid in an acetonitrile/water mixture (3:2, *v*/*v*) (solvent B). Elution was carried out using a gradient program that increased solvent B from 0 to 40%, followed by 50% after 10 min, 70% after an additional 10 min, and held for an additional 10 min. The mobile phase was delivered at a constant flow rate of 1 mL/min.

Compound identification was based on comparisons of retention times and UV–Vis absorption spectra with those of authentic reference standards, while quantification was performed using external calibration curves (0–50 mg/L). Calibration equations, coefficients of determination (R^2^), retention times, UV maxima, and LOD/LOQ values for all investigated compounds are provided in Table A1.

Method performance was evaluated through recovery experiments at three concentration levels (low, medium, high) and through intra- and inter-day precision (Table A2). Recovery values ranged between 90.8 and 99.3%, with intra-day RSD < 1% and inter-day RSD < 1.6%, indicating high accuracy and repeatability of the HPLC-DAD method.

#### 3.3.10. Preparation of Freeze-Dried Extracts

The optimal ARTP + PEF extract was freeze-dried to obtain a stable, standardized antioxidant powder. Freeze-drying was selected to eliminate solvent effects, improve storage stability, and enable accurate dosing based on extract mass. For the subsequent vinegar stability study, the optimal ARTP + PEF extract was used, as this extract exhibited the highest polyphenol concentration and antioxidant capacity among all treatments. Focusing on the most enriched extract ensured that the stability assessment reflected the performance of the technologically superior product generated through the combined ARTP + PEF process. The freeze-dried powder was stored in an airtight, light-protected container until further use.

#### 3.3.11. Stability Study of Cannabis Extract in Vinegar

To assess the stability of cannabis polyphenols in an acidic food matrix, the freeze-dried optimal ARTP + PEF extract was incorporated into vinegar and monitored over time. Only the optimal extract was used in this study, as it exhibited the highest polyphenol concentration and antioxidant capacity, representing the most technologically relevant product of the ARTP + PEF process. A structured experimental design was implemented based on the parameters provided in the vinegar stability dataset, with the independent variables and their coded levels summarized in Table 11.

The design consisted of fourteen experimental runs covering all combinations of extract concentration (X1), storage temperature (X2), and light exposure (X3), including replicated points to evaluate reproducibility. Each experimental unit consisted of 25 mL of vinegar (6% *v*/*v* acetic acid in deionized water) containing the cannabis extract at the concentration specified by the design. All samples were analyzed at five predefined time points—0, 7, 14, 21 and 28 days—to monitor the temporal evolution of antioxidant stability under each storage condition. For this purpose, a controlled stability chamber (Model CH 250, ARGO LAB, Kunshan, China) capable of maintaining precise temperature and humidity conditions, was used. The chamber was equipped with a standard LED lamp for observation, and samples assigned to the light-exposed condition were illuminated continuously (24 h) throughout the 28-day period. Storage temperature was controlled at two levels, 20 °C (ambient) and 40 °C (elevated), corresponding to the coded values used in the experimental design.

The concentration range selected for the incorporation of the freeze-dried ARTP + PEF extract into vinegar was defined at 0, 0.6, and 1.2 g/L, corresponding to the coded levels −1, 0, and +1 in Table 11. The lower level (0% *w*/*v*) served as the untreated control, allowing the intrinsic stability of vinegar to be distinguished from the contribution of the added extract. The intermediate level (0.6 g/L) was chosen as a technologically realistic concentration that provides a measurable increase in polyphenol content without affecting the physicochemical properties of the vinegar. The upper level (1.2 g/L) was included to evaluate whether higher extract loading enhances antioxidant retention during storage and to capture potential nonlinear effects of concentration on degradation kinetics. This range ensured practical relevance and sufficient variability for model development.

Total polyphenol content (TPC), ferric-reducing antioxidant power (FRAP), and DPPH radical scavenging activity were quantified at each time point. This experimental framework enabled the systematic evaluation of how extract concentration, thermal stress, and light exposure influence the chemical stability of cannabis polyphenols in vinegar. The resulting dataset supported the development of predictive models describing the combined effects of the three factors on antioxidant retention, thereby providing a mechanistic basis for assessing the suitability of the ARTP + PEF extract as a functional ingredient in vinegar-based products.

#### 3.3.12. Statistical Analysis

Statistical evaluation of the experimental data was performed using JMP Pro 16 (SAS Institute, Cary, NC, USA). Prior to model fitting, the normality of the distributions was assessed to ensure the suitability of parametric analyses. One-way ANOVA was applied to determine significant differences among treatments where appropriate. All quantitative measurements were conducted in triplicate, and extraction procedures were independently repeated to ensure reproducibility. Results are expressed as means ± standard deviations, and statistical significance was established at *p* < 0.05. Partial least squares (PLS) analysis was performed to determine variable importance (VIP) and support multivariate interpretation of ARTP parameter effects.

## 4. Conclusions

The present study demonstrated that atmospheric room-temperature plasma (ARTP) can be successfully employed as a pretreatment to enhance pulsed electric field (PEF)-assisted extraction of antioxidant compounds from *Cannabis sativa* leaves. Response surface modeling of ARTP identified the processing conditions that maximized antioxidant recovery, while HPLC analysis confirmed quantitative differences in the phenolic profile of the extract produced under the best conditions within the investigated range. The selective enhancement of protocatechuic acid and luteolin-7-*O*-glucoside provides a compositional basis for the improved antioxidant performance of the ARTP-PEF extracts. The improved extraction efficiency, together with the successful incorporation of the extract produced under the best conditions within the investigated range into vinegar, highlights the potential of the proposed sequential non-thermal process for producing antioxidant-rich ingredients suitable for functional food applications.

The combination of ARTP and PEF represents a promising green extraction strategy for the sustainable valorization of hemp leaves, contributing to the development of high-value products from an underutilized plant biomass. Although the present work was conducted at laboratory scale and evaluated a single food matrix, the encouraging results support further investigation of the proposed technology under pilot-scale conditions and in other food systems. Similar acidified matrices, including fruit juices and selected fermented beverages, may represent potential application areas; however, their suitability requires independent validation because matrix composition can substantially influence phenolic stability and antioxidant activity.

Several limitations of the present study should be acknowledged. First, the identified best-performing ARTP conditions were located partly at the boundaries of the investigated experimental domain and should therefore be interpreted as the best conditions within the studied range rather than as a global optimum. Moreover, the surface temperature of the hemp leaves was not monitored during or after the ARTP treatment. Second, the HPLC-DAD analysis was targeted to selected phenolic compounds and, given the limited number of compounds quantified above the LOQ, does not provide a comprehensive characterization of the hemp phenolic profile or allow the identification of potential plasma-induced transformation products. In addition, the mechanisms underlying the effects of ARTP on the plant matrix were not directly investigated through structural characterization or reactive-species measurements. Another important limitation is that the stability experiments were conducted in a model vinegar system (6% acetic acid), which does not contain the native phenolics, sugars, organic acids, and suspended solids present in commercial vinegar; these matrix components may influence antioxidant degradation behavior and should be examined in future studies. A further limitation is the absence of cannabinoid quantification (Δ9-THC, CBD) in both the raw leaf material and the enriched vinegar. Although the present work focused exclusively on polyphenols and antioxidant stability, cannabinoid content is essential for consumer safety and regulatory compliance, and future work will include targeted cannabinoid analysis in both the plant material and the final food matrix. Finally, although the selected condition was experimentally validated, further validation under expanded operating conditions and at pilot scale would be required to establish its robustness and industrial applicability.

Future studies should also assess the techno-economic feasibility of the process, as well as the bioaccessibility and sensory impact of hemp-derived bioactive compounds, to facilitate their successful application in functional foods. Moreover, complementary chromatographic/mass-spectrometric approaches and/or targeted optimization of extraction conditions for higher-molecular-weight flavonoids should be considered for the unidentified polyphenols.

## Figures and Tables

**Table 1:** Central Composite Design matrix for ARTP pretreatment (Coded values with actual levels in parentheses).

Run	X1–Distance (mm)	X2–Thickness (mm)	X3–Power (%)	X4–N_2_ Flow (L/min)	X5–Duration (min)	TPC (mg GAE/g dw)	FRAP (µmol AAE/g dw)	DPPH (µmol AAE/g dw)
1	1 (22)	−1 (1.5)	−1 (30)	−1 (10)	1 (10)	5.37	31.66	21.19
2	0 (17)	0 (2.5)	0 (50)	−1 (10)	0 (5.5)	7.47	34.72	28.11
3	0 (17)	0 (2.5)	0 (50)	0 (14)	−1 (1)	5.05	27.82	18.64
4	0 (17)	0 (2.5)	−1 (30)	0 (14)	0 (5.5)	6.09	35.47	18.40
5	−1 (12)	1 (3.5)	1 (70)	−1 (10)	−1 (1)	6.13	27.22	22.80
6	1 (22)	1 (3.5)	1 (70)	1 (18)	−1 (1)	5.23	22.83	18.25
7	1 (22)	0 (2.5)	0 (50)	0 (14)	0 (5.5)	3.30	10.30	14.73
8	0 (17)	0 (2.5)	0 (50)	0 (14)	0 (5.5)	4.58	22.91	19.13
9	−1 (12)	−1 (1.5)	−1 (30)	1 (18)	1 (10)	4.68	22.10	19.71
10	−1 (12)	−1 (1.5)	1 (70)	−1 (10)	1 (10)	8.47	41.78	27.40
11	0 (17)	0 (2.5)	0 (50)	0 (14)	1 (10)	6.35	17.90	19.18
12	−1 (12)	1 (3.5)	−1 (30)	−1 (10)	1 (10)	6.94	32.14	24.04
13	0 (17)	1 (3.5)	0 (50)	0 (14)	0 (5.5)	4.45	13.53	18.65
14	0 (17)	0 (2.5)	0 (50)	1 (18)	0 (5.5)	5.15	19.34	23.80
15	1 (22)	1 (3.5)	1 (70)	−1 (10)	1 (10)	5.71	26.49	24.56
16	1 (22)	1 (3.5)	−1 (30)	−1 (10)	−1 (1)	5.77	20.04	21.64
17	0 (17)	0 (2.5)	0 (50)	0 (14)	0 (5.5)	4.32	16.47	16.45
18	1 (22)	1 (3.5)	−1 (30)	1 (18)	1 (10)	6.48	22.10	22.10
19	−1 (12)	1 (3.5)	−1 (30)	1 (18)	−1 (1)	5.38	18.49	20.24
20	1 (22)	−1 (1.5)	1 (70)	1 (18)	1 (10)	4.23	12.91	18.57
21	1 (22)	−1 (1.5)	−1 (30)	1 (18)	−1 (1)	6.03	10.52	19.60
22	0 (17)	−1 (1.5)	0 (50)	0 (14)	0 (5.5)	4.17	13.58	17.84
23	−1 (12)	0 (2.5)	0 (50)	0 (14)	0 (5.5)	2.85	6.53	13.85
24	0 (17)	0 (2.5)	1 (70)	0 (14)	0 (5.5)	7.85	24.15	21.65
25	−1 (12)	−1 (1.5)	−1 (30)	−1 (10)	−1 (1)	7.04	37.63	25.30
26	−1 (12)	1 (3.5)	1 (70)	1 (18)	1 (10)	5.46	17.15	24.18
27	−1 (12)	−1 (1.5)	1 (70)	1 (18)	−1 (1)	5.29	16.89	18.48
28	1 (22)	−1 (1.5)	1 (70)	−1 (10)	−1 (1)	6.10	33.72	30.05

**Table 2:** Statistically significant model terms (*p* < 0.05) for the ARTP RSM models.

Response	Significant Terms (*p* < 0.05)	*p*-Value	Interpretation
TPC	X1^2^	0.00464	Curvature effect of distance
	X3^2^	0.00628	Curvature effect of power
	X4	0.01146	Linear effect of nitrogen flow
	X4^2^	0.04086	Curvature in nitrogen flow
FRAP	X4	0.00076	Nitrogen flow is dominant
	X1^2^	0.01265	Curvature effect of distance
	X3^2^	0.01467	Curvature effect of power
	X2 × X4	0.02699	Thickness modulates nitrogen flow
	X4^2^	0.04974	Curvature in nitrogen flow
DPPH	X4^2^	0.00002	Strong curvature effect of nitrogen flow
	X4	0.00006	Nitrogen flow is the primary driver
	X1^2^	0.00041	Curvature effect of distance
	X2 × X4	0.00323	Thickness × nitrogen flow
	X2 × X5	0.00405	Thickness × duration
	X3 × X4	0.0121	Power × nitrogen flow
	X3	0.02185	Linear effect of power
	X1 × X5	0.03343	Distance × duration
	X4 × X5	0.04716	Nitrogen flow × duration

**Table 3:** Model adequacy statistics for the ARTP response surface models.

Response	R^2^	Adj R^2^	Model F-Ratio	Model *p*-Value	Lack-of-Fit *p*-Value	RMSE	Max R^2^ *	Interpretation
TPC	0.91	0.65	3.5	0.0479	0.1679	0.7682	0.9993	Adequate model; slight curvature not fully captured but acceptable fit
FRAP	0.92	0.68	3.93	0.0354	0.585	5.115	0.9907	Good fit; no evidence of lack of fit
DPPH	0.98	0.92	16.41	0.0005	0.9183	1.102	0.9912	Outstanding fit; model captures all curvature and interactions

* Max R^2^: Theoretical maximum R^2^ achievable for the given response under the selected CCD structure, calculated from the total sum of squares and the model degrees of freedom.

**Table 4:** Optimal ARTP conditions, model predictions, validation, and comparison with controls.

Response	Optimal ARTP Conditions (*D*/*T*/*P*/*F*/*t*)	Predicted	Experimental (Mean ± SD)	*n*	% Deviation	PEF-Only Control (Mean ± SD)	No Plasma–No PEF (Mean ± SD)	Improvement vs. PEF-Only
TPC (mg GAE/g dw)	16.6/1.6/70/10/1	9.28	7.53 ± 0.21	3	−18.9%	6.12 ± 0.18	4.07 ± 0.15	23.0%
FRAP (µmol AAE/g dw)		48.69	39.18 ± 0.95	3	−19.5%	23.25 ± 0.62	18.57 ± 0.44	68.6%
DPPH (µmol AAE/g dw)		33.36	31.84 ± 0.40	3	−4.6%	21.79 ± 0.50	18.73 ± 0.38	46.1%

Values represent mean ± standard deviation (*n* = 3).

**Table 5:** Quantification of individual polyphenolic compounds (mg/g dw) under Optimal ARTP treatment compared with PEF-only and untreated controls.

Polyphenolic Compound	Optimal ARTP	PEF-Only Control	No Plasma–No PEF
Protocatechuic acid	0.54 ± 0.03 ^a^	0.44 ± 0.02 ^b^	0.31 ± 0.02 ^c^
Chlorogenic acid	<LOQ	<LOD	<LOD
Caffeic acid	<LOQ	<LOD	<LOD
Kaempferol-3-*O*-β-rutinoside	<LOQ	<LOQ	<LOQ
Luteolin-7-*O*-glucoside	0.51 ± 0.03 ^a^	0.45 ± 0.02 ^b^	0.32 ± 0.02 ^c^
Kaempferol-3-*O*-glucoside	<LOQ	<LOD	<LOD
Apigenin-7-*O*-glucoside	<LOQ	<LOD	<LOD

Values are expressed as mean ± SD (*n* = 3). Different superscript letters within the same row indicate statistically significant differences between treatments (*p* < 0.05). LOQ: limit of quantification; LOD: limit of detection.

**Table 6:** Experimental design and responses (Day 0).

Run	X1 (g/L)	X2 (°C)	X3 (Light)	TPC_0_	FRAP_0_	DPPH_0_
1	0	40	ON	–	–	–
2	0.6	20	ON	30.46	147.2	63.16
3	0.6	20	ON	31.52	188.21	73.52
4	0.6	20	OFF	30.73	169.14	82.9
5	0	40	OFF	–	–	–
6	0.6	40	OFF	30.1	155.41	56.5
7	0.6	20	ON	31.12	165.02	71.14
8	0.6	40	ON	30.38	138.89	67.45
9	1.2	20	OFF	60.27	285.67	105.57
10	0	20	OFF	–	–	–
11	1.2	20	ON	62.27	319.19	105.62
12	0	20	ON	–	–	–
13	1.2	40	ON	61.48	338.57	104.16
14	1.2	40	OFF	61.25	296.67	96.02

X1 = extract concentration, X2 = temperature, X3 = light exposure. TPC in mg GAE/L; FRAP in μmol AAE/L; DPPH in μmol AAE/L.

**Table 7:** Statistically significant model terms (*p* < 0.05) for the vinegar antioxidant responses across storage days.

Response	Day	Significant Terms (*p* < 0.05)	*p*-Value	Interpretation
TPC	0	X1	<0.0001	Extract concentration strongly increases TPC
	7	X1	0.0001	Dominant factor
	14	X1, X1 × X2	<0.0001, 0.0058	Interaction between extract concentration and temperature
	21	X1	0.0004	Stable effect across storage
	28	X1	<0.0001	Stable effect across storage
FRAP	0	X1	0.001	Extract concentration strongly increases FRAP
	7	X1	0.0011	Dominant factor
	14	X1, X2, X1 × X2, X1 × X3, X2 × X3	<0.0001, 0.0271, 0.0060, 0.0041, 0.0447	Day 14 shows the strongest interaction effects
	21	X1	0.0012	Stable effect
	28	X1	0.0012	Stable effect
DPPH	0	X1, X1 × X3	0.0029, 0.0257	Light modulates the extract effect at Day 0
	7	X1	0.0008	Dominant factor
	14	X1	0.0008	Stable effect
	21	X1	0.0002	Maximum DPPH at Day 21
	28	X1	0.0011	Stable effect

**Table 8:** Model adequacy statistics for the vinegar antioxidant responses across storage days.

Response	Day	R^2^	Adj R^2^	RMSE	Lack-of-Fit *p*-Value	Interpretation
TPC	0	0.9996	0.9987	0.57	0.3515	Excellent fit; no lack-of-fit
	7	0.9957	0.9871	1.48	0.0854	Very good fit; borderline LOF but acceptable
	14	0.9987	0.9961	0.67	0.5371	Excellent fit
	21	0.9914	0.9743	1.81	0.4949	Very good fit
	28	0.997	0.9909	1.04	0.3251	Excellent fit
FRAP	0	0.9839	0.9516	17.47	0.7245	Very good fit
	7	0.9852	0.9555	13.47	0.4375	Very good fit
	14	0.999	0.9969	3.26	0.8083	Outstanding fit
	21	0.9834	0.9503	14.1	0.282	Very good fit
	28	0.9832	0.9495	13.54	0.3762	Very good fit
DPPH	0	0.9701	0.9102	5.66	0.3785	Good fit
	7	0.9895	0.9685	5.44	0.557	Excellent fit
	14	0.9882	0.9645	6.51	0.1438	Very good fit
	21	0.9952	0.9857	3.88	0.6618	Outstanding fit
	28	0.9857	0.9571	6.81	0.5166	Excellent fit

**Table 9:** Overall stability behavior of cannabis vinegar during 28-day storage.

Component	Description	Summary
Stability trend of TPC	Change from Day 0 → Day 28	Moderate decrease (~10–20%); high stability across all treatments
Stability trend of FRAP	Change from Day 0 → Day 28	Strong decrease (~20–40%); thermal degradation more pronounced at 40 °C
Stability trend of DPPH	Change from Day 0 → Day 28	Polyphasic pattern: increase → peak at Day 21 → decline
Effect of X1 (extract concentration)	Influence on stability	Dominant stabilizing factor; higher X1 maintains higher antioxidant levels on all days
Effect of X2 (temperature)	Influence on stability	40 °C accelerates FRAP and DPPH degradation; minimal effect on TPC
Effect of X3 (light exposure)	Influence on stability	Minor oxidative effect; less impactful than temperature
Most stable condition	Factor combination	X1 = 1.2 g/L, X2 = 20 °C, X3 = OFF (dark)
Least stable condition	Factor combination	X1 = 0 g/L, X2 = 40 °C, X3 = ON (light)
Overall interpretation	General conclusion	Cannabis vinegar shows good antioxidant stability; extract concentration is the key determinant of preservation

**Table 10:** Actual values and coded levels of the independent variables used for ARTP optimization.

Independent Variables	Coded Units	Coded Levels		
		−1	0	1
Distance (*D*, mm)	*X*_1_	12	17	22
Thickness (*T*, mm)	*X*_2_	1.5	2.5	3.5
Power (*P*, %)	*X*_3_	30	50	70
Nitrogen flow (*F*, L/min)	*X*_4_	10	14	18
Duration (*t*, min)	*X*_5_	1	5.5	10

**Table 11:** Independent variables and coded levels used in the vinegar stability study.

Independent Variables	Coded Units	Coded Levels		
		−1	0	1
Extract concentration (g/L)	*X*_1_	0	0.6	1.2
Temperature (°C)	*X*_2_	20	—	40
Light exposure	*X*_3_	OFF	—	ON

## Data Availability

All related data and methods are presented in this paper. Additional inquiries should be addressed to the corresponding authors.

## Appendix A

**Table A1:** Equations of calibration curves and LOD and LOQ for each compound were identified through HPLC-DAD.

Polyphenolic Compound	Equation (Linear)	R^2^	RT (min)	UVmax (nm)	LOD (mg/L)	LOQ (mg/L)
Protocatechuic acid	y = 21,282.98x − 63,918.97	0.977	13.40	260	7.47	22.64
Chlorogenic acid	y = 50,320.40x − 23,038.36	0.994	21.95	325	3.67	11.11
Caffeic acid	y = 937,658.95x + 12,216.24	0.999	24.77	322	0.75	2.29
Kaempferol-3-*O*-β-rutinoside	y = 35,702.88x + 9620.15	0.998	32.68	344	2.15	6.51
Luteolin-7-*O*-glucoside	y = 34,875.94x − 16,827.36	0.999	35.95	347	1.28	3.89
Kaempferol-3-*O*-glucoside	y = 50,916.85x − 42,398.80	0.996	38.72	265	3.00	9.08
Apigenin-7-*O*-glucoside	y = 64,742.65x + 15,897.94	0.998	39.85	336	2.22	6.72

**Table A2:** Recovery and precision data for HPLC-DAD quantification of polyphenolic standards.

Polyphenolic Compound	Recovery Low (%)	Recovery Medium (%)	Recovery High (%)	Intra-Day RSD (%)	Inter-Day RSD (%)
Protocatechuic acid	92.4	96.8	98.1	0.85	1.42
Chlorogenic acid	92.3	97.7	98.8	0.34	0.91
Caffeic acid	93.1	95.9	99.2	0.72	1.18
Kaempferol-3-*O*-β-rutinoside	90.8	95.9	98.0	0.66	1.25
Luteolin-7-*O*-glucoside	94.2	97.5	99.3	0.58	1.36
Kaempferol-3-*O*-glucoside	91.7	96.1	98.4	0.69	1.48
Apigenin-7-*O*-glucoside	92.1	96.4	98.7	0.74	1.52
